# Management of Lumbar Hernia Secondary to Retroperitoneal Abscess Drainage

**DOI:** 10.7759/cureus.83220

**Published:** 2025-04-29

**Authors:** Shadi Al-Bahri, Zoha Khalid

**Affiliations:** 1 Department of Surgery, Sheikh Tahnoon Medical City, Al Ain, ARE; 2 Department of Surgery, College of Medicine and Health Sciences, United Arab Emirates University (UAEU), Al Ain, ARE; 3 Department of Surgery, Burjeel Hospital, Al Ain, ARE

**Keywords:** adrenalectomy, hernia mesh, interventional radiology drainage, lumbar hernia, open hernia repair

## Abstract

Lumbar hernia is a rare defect that develops through the posterolateral abdominal wall. It can be classified as either congenital or acquired and may occur secondary to traumatic, iatrogenic, or infectious etiologies. Surgical management is the standard approach, with laparoscopic techniques emerging as the preferred method.A 47-year-old woman presented with swelling and pain in the left flank several months after undergoing a left laparoscopic adrenalectomy, which resulted in an abscess formation requiring interventional radiologic (IR) drainage. A computed tomography (CT) scan confirmed a left lumbar hernia, and an open mesh repair was performed. Due to their rare presentation, lumbar hernias do not have a standardized treatment algorithm. Both open and laparoscopic approaches can be utilized, but mesh repair is usually required to prevent hernia recurrence. Considering the surrounding musculature and bony prominences, knowledge of the anatomy is key to a successful and durable repair.* *Lumbar hernias should be considered a potential complication arising from drain placement in the flank. Surgical repair is appropriate for symptomatic lumbar hernias. The choice between laparoscopic and open approaches is made based on various patient-related considerations.

## Introduction

Lumbar hernias are a rare abdominal wall pathology, comprising less than 1.5% of abdominal wall defects [1]. They were first reported by Dr. De Garangeot in 1731 when he described the hernia in the lumbar region during an autopsy [2]. The etiology can be classified as congenital or acquired. Anatomically, lumbar hernias can develop in the flank within the superior lumbar triangle, known as Grynfeltt-Lesshaft's triangle, or the inferior lumbar triangle, known as Petit’s triangle.

Lumbar hernias can be asymptomatic, but when symptomatic, their presentation remains similar to other abdominal wall defects. This includes swelling in the posterolateral abdominal wall in the flank and discomfort on exertion, which is usually spontaneously reducible [3]. Inadequate exposure to this clinical entity may lead to delays in diagnosis and management, potentially leading to bowel compromise or more complicated repair. Because lumbar hernias have a tendency to progress, delayed diagnosis is associated with increased morbidity [4]. Computed tomography (CT) scan is the investigation of choice [5,6], and the preferred treatment is surgical repair [4] through an open or laparoscopic approach. This case is in line with the SCARE 2020 criteria [7].

## Case presentation

A 47-year-old woman presented with pain and discomfort in the left lumbar region for several months, worsening with activity and coughing. The swelling had an insidious onset but had gradually progressed, leading to significant discomfort. She is diabetic and has adrenal hypertension, Cushing’s syndrome, and vitamin D deficiency. Her past surgical history is significant for a left laparoscopic adrenalectomy for a 4 cm adrenocortical adenoma performed 14 months prior to her presentation. Postoperatively, she developed a retroperitoneal abscess, which was drained. Physical examination revealed left flank swelling with mild tenderness and no overlying skin changes.

**Figure 1:**
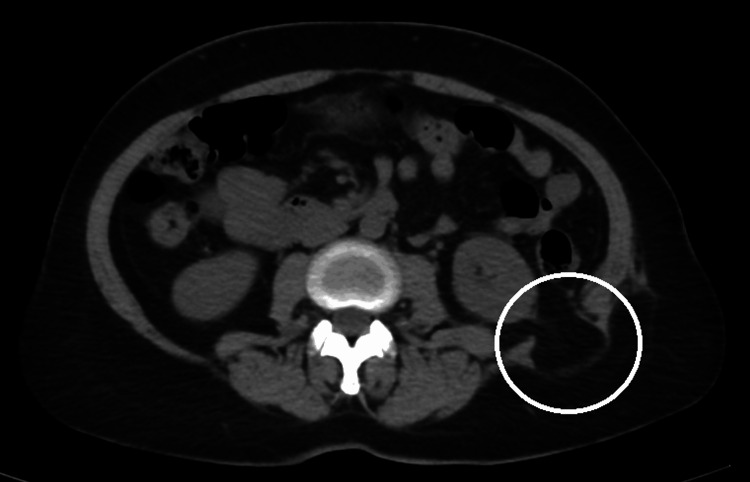
Cross-sectional CT images showing a lumbar hernia defect delineated by the circle, with herniating retroperitoneal fat and associated surrounding musculature. CT: computed tomography.

A CT scan of the abdomen and pelvis showed a 2.7 cm defect in the superior left lumbar triangle (Figure 1). The contents of the hernia sac were limited to retroperitoneal fat, with no surrounding muscular atrophy noted. A diagnosis of left-sided lumbar hernia was made, and the patient consented to an open mesh repair, although the laparoscopic approach was considered.

**Figure 2:**
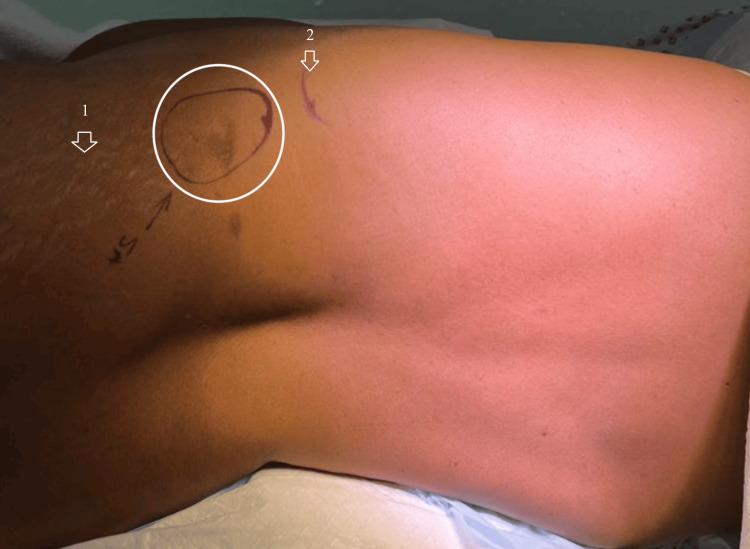
Patient is positioned in the right lateral decubitus position with flexing of the table at the hip to allow for expansion of the space between the bony prominences separating the subcostal margin (marked with arrow 2) from the iliac crest (marked with arrow 1). Encircled is the site of the lumbar hernia.

**Figure 3:**
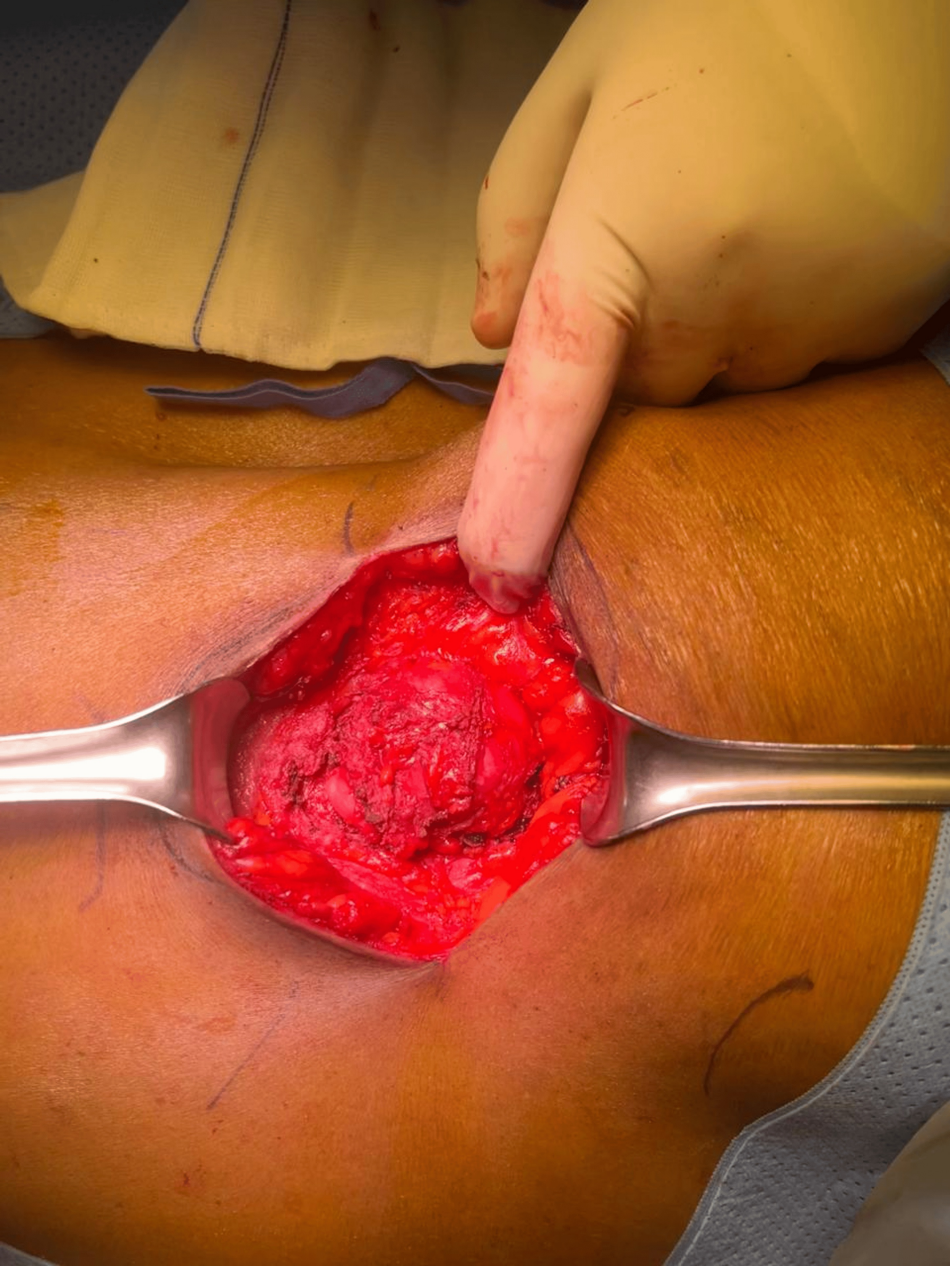
Identification of the hernia sac surrounded by the flank muscles, the external oblique, internal oblique, and transverse abdominis.

The author obtained consent from the patient and proceeded with the operation. After administering general anesthesia, the patient was placed in the right lateral position (Figure 2), and a transverse incision was made over the defect. Dissection was performed until the transversalis fascia and hernia sac were identified and circumferentially dissected from the overlying flank muscles (Figure 3). Next, the retroperitoneum was accessed by separating it from the overlying transversalis fascia from the 12th rib superiorly to the posterior rectus sheath medially, inferiorly to the iliac crest, and laterally to the psoas muscle. A 20x25 cm polypropylene mesh (Ultrapro, Ethicon, USA) was sutured to the 12th rib, avoiding the neurovascular structures. It was then affixed medially to the posterior rectus sheath at the semilunar line, inferiorly to the iliac crest, and laid laterally superior to the psoas muscle without fixation at that site to avoid the overlying nerves. The muscle layers were then closed. There were no perioperative complications.

## Discussion

Congenital lumbar hernias comprise 20% of the lumbar hernias identified in clinical practice [2] and are often associated with other musculoskeletal deformities but can include anomalies of the kidneys and spinal meninges [7]. Acquired lumbar hernias are more common, accounting for up to 80% of cases [2], and are further divided into primary and secondary. Primary acquired lumbar hernias develop spontaneously, whereas secondary lumbar hernias develop in response to trauma, infections, or iatrogenic causes. Iatrogenic causes of lumbar hernias have been described in the literature, including flank incisions [8], hip [9], spine surgery [10], and laparoscopic procedures [11].

Alternatively, the classification of lumbar hernias is described by the two lumbar triangles, which are regions in the flank where muscles overlap, creating the potential for hernia development. The superior lumbar triangle is bordered superiorly by the 12th rib, medially by the erector spinae, and laterally by the internal oblique. The borders of the inferior lumbar triangle are the iliac crest inferiorly, external oblique laterally, and latissimus dorsi medially [12].

Our case of superior triangle lumbar hernia presented post-IR drainage done for a flank abscess complicating laparoscopic adrenalectomy. Abscesses have a weakening effect on the surrounding tissue [13], and catheter insertion for abscess drainage likely further weakens the musculature, preventing natural healing and scarring, resulting in the development of a lumbar hernia at that location.

Clinically, lumbar hernias can manifest from asymptomatic to symptomatic to complicated. Symptomatic patients will present with a wide spectrum of complaints ranging from a palpable swelling to bowel incarceration or even strangulation leading to bowel ischemia or perforation [14].

CT is the investigation of choice in confirming a diagnosis of lumbar hernia, as it provides precise information about the location, size, and contents of the defect. CT also differentiates a hernia from a soft tissue mass and identifies the condition of the surrounding muscles, including muscular atrophy [2,15,16]. A complete diagnosis following CT helps determine the most appropriate surgical approach [17] and considers the appropriate mesh characteristics to be used.

As a rarely encountered condition, lumbar hernias do not have a standardized management algorithm [16]. In addition to the associated symptoms, the hernia defect may also increase in size, adding another layer of complexity to surgical repair [4]. Another challenge faced by surgeons is the anatomy of the lumbar region. Therefore, preoperative revision of the associated anatomy is crucial [18].

For small fascial defects, approximating the surrounding musculature may be sufficient [3]. However, primary repairs tend to have high tension, especially with the increasing size of the defect [19]. Larger lumbar hernias require a fascial flap or mesh placement [3]. However, synthetic mesh is used more routinely as fascial flaps necessitate extensive dissection, risking flap ischemia [12,19].

Surgical options include open and laparoscopic approaches, such as the transabdominal and totally extraperitoneal approach. Laparoscopic repair is now being more routinely performed [13] because it allows for better visualization of the defect and surrounding structures, avoiding dissection through the abdominal wall [18]. Acquired defects tend to have altered surrounding anatomy, so laparoscopy can yield better results than open repair [12]. The open approach requires a larger incision and extensive dissection, increasing the risk of postoperative pain and complications [18]. An open or laparoscopic extraperitoneal approach is appropriate for primary hernias as they are usually small with normal surrounding anatomy [16]. Defects larger than 15 cm [16], complicated hernias, and failed laparoscopies would require open repairs [19].

The rationale for utilizing the open approach in our case was the recent access to the extraperitoneal plane during the laparoscopic adrenalectomy. Given the development of an abscess at the site and the left kidney adjacent to the defect, it was felt that avoiding the extraperitoneal plane was ideal to avoid injury to the surrounding structures. The open approach offered the advantage of approaching the hernia sac through undisturbed planes without scarring or adhesions. We were aware of the advantages posed by utilizing a laparoscopic transabdominal approach, but after discussing the risks and benefits of the different approaches with the patient, the open approach was chosen.

## Conclusions

We present this case to shed light on the possibility of developing secondary acquired lumbar hernias through interventions that may initially be considered benign, such as the placement of a drain by interventional radiology. Early intervention is ideal to avoid hernia complications if there are no contraindications to repair. Although there is currently no standardized approach to lumbar hernias, surgical correction is the appropriate management for symptomatic hernias, and a laparoscopic approach appears to offer more advantages than open repair. A thorough review of lumbar anatomy is essential for safe lumbar hernia repairs, given their rarity. 
